# Gaps in the Outpatient Referral Cascade for Patients With Medicaid

**DOI:** 10.1001/jamanetworkopen.2025.37047

**Published:** 2025-10-09

**Authors:** Parsa Erfani, Nina Jain, Yu-Hui Chen, Dale S. Adler, Mallika L. Mendu

**Affiliations:** 1 Department of Medicine, Brigham and Women’s Hospital, Boston, Massachusetts; 2 Harvard Medical School, Boston, Massachusetts; 3 Department of Data Science, Dana-Farber Cancer Institute, Boston, Massachusetts; 4 Department of Cardiology, Brigham and Women’s Hospital, Boston, Massachusetts; 5 Department of Medicine, Renal Division, Brigham and Women’s Hospital, Boston, Massachusetts

## Abstract

This cohort study examines whether Medicaid status is independently associated with outpatient referral scheduling and completion at a large, multisite academic medical center.

## Introduction

Patients with Medicaid coverage have low rates of outpatient referrals scheduled and completed.^1,2,3,4,5^ It is unclear whether this disparity arises from insurance-related obstacles or care delivery barriers linked to comorbidities and demographic characteristics, such as income, education, and language. We aimed to determine whether Medicaid status is independently associated with outpatient referral scheduling and completion at a large, multisite academic medical center.

## Methods

This observational cohort study follows STROBE reporting guidelines. This study was determined to be exempt from review by the Mass General Brigham institutional review board. Informed consent was not required as the study posed minimal risk to participants. We utilized electronic health record data to identify all internal referrals (originating from outpatient and inpatient departments at Mass General Brigham [MGB]) to 39 medical and surgical ambulatory clinics at Brigham and Women’s Hospital in 2022. The primary exposure was insurance status at the time of referral, and primary outcomes were referral scheduling proportion (referrals scheduled divided by total referrals) and referral completion proportion (referrals with completed visit divided by total referrals) (eMethods in Supplement 1).

Generalized estimating equation logistic regression models evaluated associations between insurance status and scheduling or completion proportions, adjusting for age, sex, race, ethnicity, language of care, education level, income, and Charlson Comorbidity Index score. Regression models were repeated with Medicaid disaggregated into 2 subgroups (MGB Medicaid accountable care organization [ACO] and non-MGB Medicaid plans). Models for scheduling were also repeated per recipient clinic (excluding 6 low-volume clinics). Adjusted odds ratios (aORs) were calculated, and statistical significance was defined as a 95% CI that did not cross the null value of 1. Data were analyzed with SAS statistical software version 9.4 (SAS Institute).

## Results

**Figure 1.**
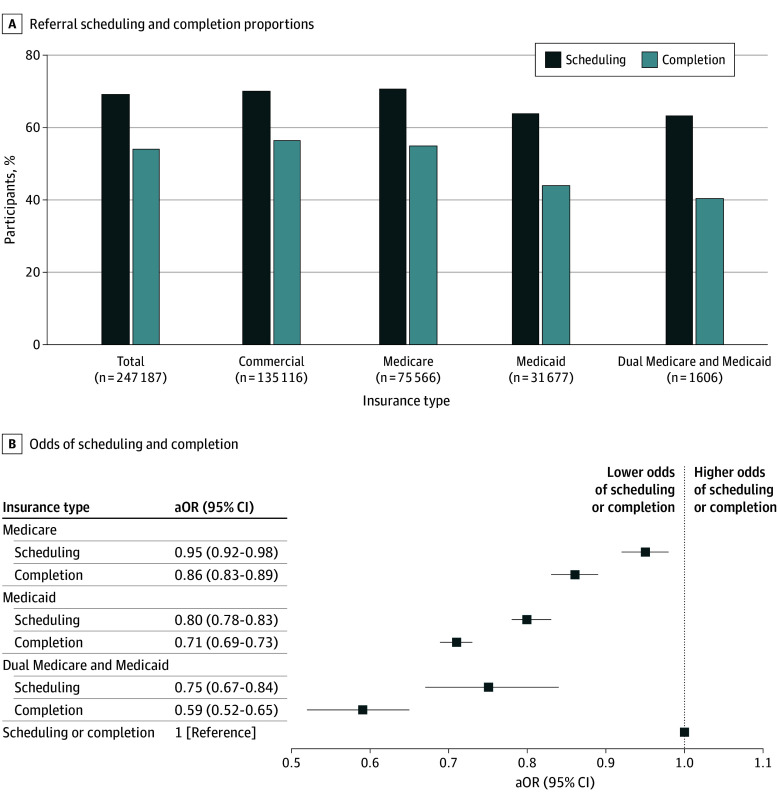
Referral Scheduling and Completion, by Insurance Graphs show scheduling and completion proportions (A) and adjusted odds ratios (aORs) for scheduling and completion (B) in 2022. Regression was adjusted for age, sex, race, ethnicity, language of care, education level, income, and Charlson Comorbidity Index score. Other insurance category is not shown because of insurance heterogeneity and low numbers.

Of the 247 187 referrals, the majority were for patients aged 50 years and older (147 413 referrals [59.6%]), English speaking (227 600 referrals [92.8%]), and with a Charlson Comorbidity Index score of 0 (190 777 referrals [77.2%]). Insurance types were commercial (135 116 referrals [54.7%]), Medicare (75 566 referrals [30.6%]), Medicaid (31 677 referrals [12.8%]), and dual Medicare and Medicaid (1606 referrals [0.6%]). Across all referrals, 69.2% (171 073 referrals) were scheduled and 54.1% (133 805 referrals) were completed (Figure 1A). After adjusting for potential confounding factors in a multivariable regression (243 769 referrals), Medicaid patients had significantly lower odds of having a scheduled appointment (aOR, 0.80; 95% CI, 0.78-0.83) or completed visit (aOR, 0.71; 95% CI, 0.69-0.73), compared with commercially insured patients. Dual-enrolled Medicare and Medicaid patients had lower odds of both scheduling (aOR, 0.75; 95% CI, 0.67-0.84) and completion (aOR, 0.59; 95% CI, 0.52-0.65) (Figure 1B).

**Figure 2.**
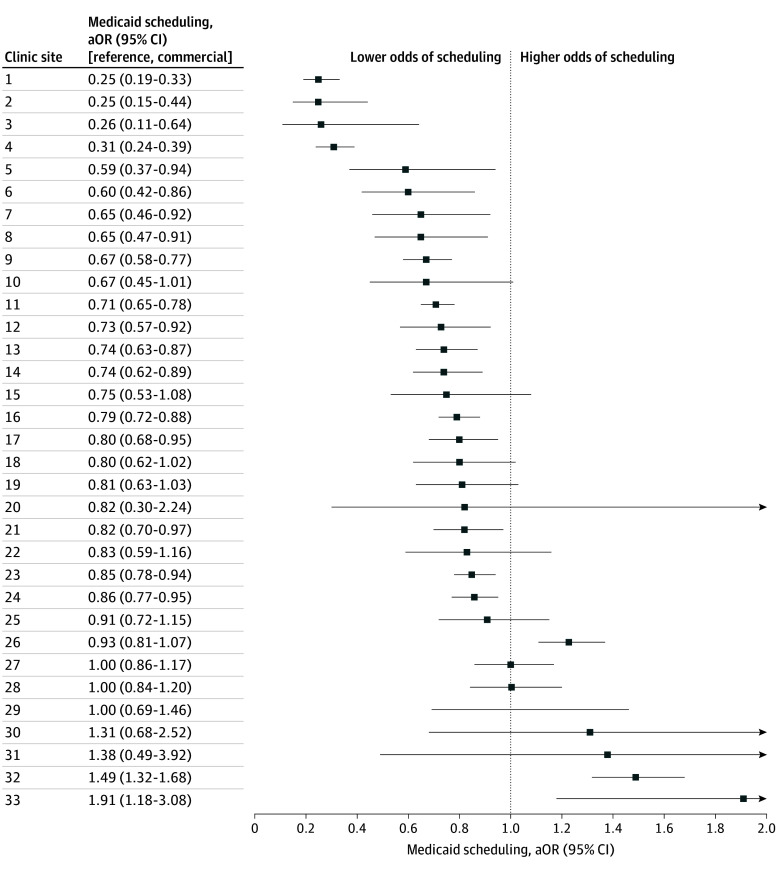
Referral Scheduling for Medicaid vs Commercial Insurance, by Clinic Data for 2022 are shown. Regression was adjusted for age, sex, race, ethnicity, language of care, education level, income, and Charlson Comorbidity Index score. aOR indicates adjusted odds ratio.

In a Medicaid subgroup analysis, patients in the MGB Medicaid ACO had modestly lower odds of referral scheduling (aOR, 0.92; 95% CI, 0.89-0.95) compared with commercially insured patients, whereas patients with non-MGB Medicaid plans had markedly lower odds (aOR, 0.61; 95% CI, 0.58-0.64). Both groups had lower odds of referral completion (MGB Medicaid ACO, aOR, 0.78; 95% CI, 0.75-0.80; non-MGB Medicaid plans, aOR, 0.57; 95% CI, 0.54-0.60). Individual clinic-level assessment revealed substantial variation in scheduling aORs for Medicaid (range, 0.25-1.91) and dual-enrolled patients (range, 0.06-3.16) (Figure 2).

## Discussion

This cohort study found that patients with Medicaid coverage face barriers scheduling outpatient referral visits after accounting for other factors impacting access. Barriers were most evident for dual-enrolled patients and those in non-MGB Medicaid plans. The latter may face network coverage barriers when referred from emergency departments or inpatient settings. These findings highlight how segmented Medicaid ACOs may contribute to poor referral outcomes for Medicaid patients when they seek care. Variations in Medicaid scheduling across clinics within the same health system also suggest that operational factors (eg, referral triage processes) likely impact patient experience.

Lower access to specialty care is associated with poor health outcomes and high hospitalization rates.^6^ State governments and health systems may improve care and reduce downstream health expenditures for Medicaid patients by designing interventions that improve their referral outcomes. Valuable interventions could include converting referrals to external practitioners when patients face network coverage barriers or creating centralized scheduling systems to reduce clinic-level variations in patient experience. Although these findings reflect gaps within a single academic health system, they likely highlight broader structural issues in Medicaid referral networks across the US. Limitations include the inability to capture scheduled appointments not linked to referrals, to track whether referrals were completed elsewhere, and to measure time interval between referral and appointment (which may impact completion and differ by insurance).

## Notes

Supplement 1. eMethods

Supplement 2. Data Sharing Statement
