# Supplementary material for: Gaps in the Outpatient Referral Cascade for Patients With Medicaid
Source: JAMA Netw Open. 2025 Oct 9;8(10):e2537047. doi: 10.1001/jamanetworkopen.2025.37047 (PMC12511988; doi:10.1001/jamanetworkopen.2025.37047)
Supplement: Supplement 1. — eMethods [file jamanetwopen-e2537047-s001.pdf]

Supplemental Online Content

Erfani P, Jain N, Chen YH, Adler DS, Mendu ML. Gaps in the outpatient referral cascade for patients with Medicaid. *JAMA Netw Open*. 2025;8(10):e2537047. doi:10.1001/jamanetworkopen.2025.37047

eMethods

This supplemental material has been provided by the authors to give readers additional information about their work.

© 2025 Erfani P et al. *JAMA Network Open*.

eMethods

Scheduling and completion proportions were calculated as the proportion of referrals placed in 2022 that resulted in a scheduled appointment and a completed visit by December 2023, respectively, allowing for at least a one-year lag time. Descriptive statistics were used to characterize the patients at referral. All patient characteristics were converted to categorical variables. An unknown category was created for missing data of greater than 1% for a given covariate. Percentage of missing data ranged from 6-12% for variables (Table). Missing data less than 1% was excluded from the analysis. Patients with age <18 years old (n=1,541) were excluded from the analysis of education level. Race, ethnicity, and education level are self-reported. Median household income by zip code was assigned to participants as the estimated income.

As some patients had multiple referrals, generalized estimating equation (GEE) logistic regression was used to assess the relationship between patient characteristics and scheduling as well as completion of an appointment accounting for correlation between referrals within the same subjects. Univariable analysis was performed and the variables included in the multivariable model (age, sex, race, ethnicity, insurance status, language of care, education level, income, and Charlson Comorbidity Index [CCI]) were chosen based on the results from the univariable analysis. Insurance status was defined as the primary insurer at time of referral. Insurance status categories included Commercial, Medicare, Medicaid, Medicare/Medicaid, and Other. Medicaid plan type categories included MGB Medicaid accountable care organization and non-MGB Medicaid plans.

Table. Percent missing data for relevant variable

	**No. Referrals (%)**
**All Referrals**	247,187 (100%)
Race - Unknown	30,187 (12.2%)
Ethnicity - Unknown	14,378 (5.8%)
Education Level - Unknown	20,560 (8.4%)
Charlson comorbidity index - Unknown	15,000 (6.1%)
Income - Unknown	17,850 (7.3%)

© 2025 Erfani P et al. *JAMA Network Open*.
