# Supplementary material for: Gaps in the Outpatient Referral Cascade for Patients With Medicaid
Source: JAMA Netw Open. 2025 Oct 9;8(10):e2537047. doi: 10.1001/jamanetworkopen.2025.37047 (PMC12511988; doi:10.1001/jamanetworkopen.2025.37047)
Supplement: Supplement 2. — Data Sharing Statement [file jamanetwopen-e2537047-s002.pdf]

Data Sharing Statement

Erfani. Gaps in the Outpatient Referral Cascade for Patients With Medicaid. *JAMA Netw Open*. Published October 09, 2025. doi:10.1001/jamanetworkopen.2025.37047

Data
Data available: No

Additional Information
Explanation for why data not available: This study utilized data from institutional repositories. Due to the inclusion of protected health information (PHI), these data are not publicly available and cannot be shared outside the institution.
